# Wnt signalling in the articular cartilage: A matter of balance

**DOI:** 10.1111/iep.12472

**Published:** 2023-02-26

**Authors:** Amandeep Kaur Gill, Peter J. McCormick, David Sochart, Giovanna Nalesso

**Affiliations:** ^1^ Centre for Endocrinology, William Harvey Research Institute, Barts and the London School of Medicine, Queen Mary University of London London UK; ^2^ South West London Elective Orthopaedic Centre Epsom UK; ^3^ Department of Comparative Biomedical Sciences, School of Veterinary Medicine University of Surrey Guildford UK

**Keywords:** articular cartilage, osteoarthritis, Wnt

## Abstract

Degradation of the articular cartilage is a hallmark of osteoarthritis, a progressive and chronic musculoskeletal condition, affecting millions of people worldwide. The activation of several signalling cascades is altered during disease development: among them, the Wnt signalling plays a pivotal role in the maintenance of tissue homeostasis. Increasing evidence is showing that its activation needs to be maintained within a certain range to avoid the triggering of degenerative mechanisms. In this review, we summarise our current knowledge about how a balanced activation of the Wnt signalling is maintained in the articular cartilage, with a particular focus on receptor‐mediated mechanisms.

## 1 OSTEOARTHRITIS

Osteoarthritis (OA) is a chronic and degenerative musculoskeletal condition, affecting 16% of the worldwide population over the age of 15.^1^ From a clinical point of view, OA is characterised by progressive and irreversible degeneration of the articular cartilage (AC), inflammation of the synovial lining and abnormal subchondral bone remodelling.^2^ Osteoarthritis is a multifactorial disease: ageing, genetic predisposition, history of trauma and co‐morbidities, such as diabetes or hypercholesterolemia, have been associated with the onset of OA or the exacerbation of its symptoms and clinical features.^3^, ^4^, ^5^

No pharmacological treatment can revert the progression of the disease, and pain relief is the main therapeutic option for patients. When this does not suffice anymore and joint mobility is completely compromised, the patient is offered a joint replacement surgery. This option is not devoid of potential post‐operative complications, especially in the most elderly patients. Furthermore, in 30% of patients, joint replacement with a prosthesis does not resolve chronic pain, thereby only partially improving patients' quality of life.^6^

## 2 THE ARTICULAR CARTILAGE

The AC is the connective tissue covering the edges of the bones in the diarthrodial joints. The tissue allows a smooth diarthrosis being elastic and conferring resistance to compression upon the joint. Its main constituents are a thick extracellular matrix (ECM), made of proteoglycans and collagens, and only one cell type, the chondrocyte. Proteoglycans are negatively charged molecules providing the AC with its high resistance to compressive loads by attracting water into the tissue. Collagens are organised in fibres conferring the AC tensile strength.^7^ Chondrocytes are responsible for the turnover of ECM components by expressing and secreting both ECM components and the enzymes responsible for their degradation, such as metalloproteases (MMPs). As the AC is nonvascularised and noninnervated, the tissue relies on the subchondral bone and the synovial fluid to receive nutrients and to maintain the necessary tissue lubrication.^8^, ^9^

## 3 DEGRADATION OF THE ARTICULAR CARTILAGE IN OSTEOARTHRITIS

During the development of OA, the metabolic balance in the AC skews towards catabolism. The expression and activity of MMPs, such as MMP3 and MMP13, and A Disintegrin And Metalloproteinase with Thrombospondin Motifs (ADAMTs) enzymes, such as ADAMTS4 and ADAMTS5 are strongly upregulated in the AC along disease progression.^10^ Conversely, the expression of some tissue inhibitors of metalloproteases (TIMPs) is reduced.^11^ Metalloproteases cut and degrade proteoglycans and collagens, leading to tissue erosion and to the onset of the disease.

Increasing evidence suggests that OA is an abnormal recapitulation of osteogenesis. Bones are derived from the cartilage anlagen: chondrocytes in the anlage undergo a coordinated process of proliferation and maturation which culminates in hypertrophic maturation. Hypertrophic chondrocytes (HC) can then indirectly and directly contribute to bone formation. In the first case, HC undergo terminal differentiation, driving the secretion of mineralised cartilage and blood vessels invasion, after which they die through apoptosis. Vascular invasion allows the arrival of blood vessel‐associated pericytes, osteoclasts and bone progenitor cells in the anlage, promoting bone formation. Hypertrophic chondrocytes can also transdifferentiate into osteoblasts, therefore directly generating bone tissue.^12^, ^13^, ^14^

Differently, synovial joints originate from the condensation of mesenchymal cells in structures called interzones. Interzone cells directly differentiate into articular chondrocytes, which do not undergo hypertrophic differentiation.^15^

During the development of OA, deregulation of several signalling cascades can lead articular chondrocytes to restart the differentiation process from where it had been interrupted during joint development and undergo hypertrophic maturation and calcification.^16^ These phenomena contribute to the loss of elasticity and resistance to compression of the ECM, leading to tissue destruction and ultimately compromising joint mobility and inducing pain.^16^

## 4 THE WNT SIGNALLING

Deregulation of the Wnt signalling plays a major role in the development and progression of OA. Wnts are a family of 19 glycosylated and lipid‐modified ligands activating a complex network of signalling pathways. To activate any of these pathways, Wnts engage with the Frizzled (FZD) family of receptors. The Wnt/FZD complexes associate with different co‐receptors, leading to the initiation of specific intracellular signalling. The association of FZDs with the Low‐density Lipoprotein‐Related Protein 5 or 6 (LRP5/6) receptors leads to the destabilisation of a multiprotein ‘destruction complex' which includes among its members Axin2, Adenomatous polyposis coli (APC), Glycogen Synthase Kinase 3 (GSK3), Casein Kinase 1 (CK1), protein phosphatase 1A (PP2A) and the E3‐ubiquitinin ligase beta‐transducin repeat‐containing protein (β‐TrCp). In the absence of an activating stimulus, the complex traps β‐catenin within the cytoplasm and addresses it for ubiquitin‐mediated degradation.^17^ The disaggregation of the destruction complex promotes the accumulation of β‐catenin which can then translocate to the nucleus where it binds to components of the T‐cell factor/lymphoid enhancer factor (TCF/LEF) family of proteins, promoting the transcription of cell and context‐specific genes.^18^ Among these, Axin2 seems to be a well‐preserved target across cell types and species.^19^

Frizzled, however, have also been reported to associate with other co‐receptors, such as the Receptor Tyrosine Kinase‐like Orphan Receptor 2 (ROR2) and the Related to Receptor Tyrosine Kinase (RYK),^20^, ^21^ promoting the activation of less characterised signalling cascades. These have been grouped under the name of β‐catenin‐independent pathways. Their triggering does not promote the accumulation of β‐catenin but leads instead to the activation of other downstream targets such as Calcium Calmodulin Kinase II (CaMKII),^22^, ^23^ Protein Kinase A (PKA) and Protein Kinase C (PKC).^24^, ^25^

## 5 THE WNT SIGNALLING IN OA

The Wnt/β‐catenin‐dependent signalling is the most characterised of the Wnt pathways at molecular level. The activation of this pathway is required and sufficient for synovial joint formation;^26^ however, it needs to be tightly regulated in a time‐ and space‐dependent manner to allow the integrity of the tissue to be maintained in the adult life.^27^, ^28^

Overactivation of the Wnt/β‐catenin signalling has indeed been associated with degradation of the AC in OA. Polymorphisms in genes expressing components and/or modulators of this pathway have been linked to a higher risk of developing OA. The Arg200Trp and the Arg324Gly substitutions in the secreted Frizzled‐Related Protein 3 (sFRP3), a soluble scavenger of Wnt ligands, lead to loss‐of‐function mutations which have been reported to be more frequent in patients with clinical signs of OA.^29^, ^30^, ^31^ sFRP3‐deficient mice developed more severe cartilage destruction both in a model of inflammatory arthritis and in a surgically induced OA model.^32^, ^33^ In addition, the expression of Dikkopf1 (Dkk1), an inhibitor of the Wnt/β‐catenin pathway, is lower in the AC of OA patients in comparison to  healthy donors.^34^ Increased expression of β‐catenin has been detected in the AC of OA patients and of mice in which OA was surgically induced.^35^, ^36^, ^37^ However, overactivation of the Wnt/β‐catenin pathway in the tissue both through genetic manipulation or pharmacological modulation did not conclusively determine a cause–effect link between the activation of this branch of the Wnt signalling and tissue degeneration. Genetic activation of the β‐catenin gene in the AC promoted OA development in mice. This was associated with increased expression of MMP13 and ADAMTS5, upregulation of hypertrophic markers such as Collagen Type X (ColX) and increased cell death, both in the temporomandibular joint (TMJ) and in the knee joint.^38^, ^39^ An additional study from Cai et al. showed that mechanical stress can promote TMJ OA via overactivation of the Wnt/β‐catenin signalling.^40^

However, loss of β‐catenin transcriptional activity was also shown to disrupt tissue homeostasis and promote the development of OA‐like lesions in the AC. Overexpression of the inhibitor of β‐catenin and TCF‐4 (ICAT) led to reduced chondrocyte proliferation, increased apoptosis and reduced skeletal grown after birth in mice.^41^ These mice also developed spontaneous OA with ageing.^42^ Downregulation of β‐catenin in the superficial cells of the AC also induced downregulated expression of lubricin (PRG4), an important joint lubricant, and OA‐like cartilage degeneration. Indeed, modulation of PRG4 expression at mRNA level has been shown to be linked to the activation of the Wnt/β‐catenin signalling in isolated chondrocytes.^43^

These data suggest that a tight regulation of the Wnt/β‐catenin signalling in space and time within the AC is required to maintain tissue homeostasis; however, the mechanisms regulating this balanced activation remain largely uncharacterised.

## 6 THE WNT/β‐CATENIN‐INDEPENDENT SIGNALLING NETWORK IN THE AC

The role of the Wnt/β‐catenin‐independent pathways in the AC and OA is far less characterised. Our current knowledge is mainly derived from developmental studies.

The Wnt5a/ROR2 pathway has an important homeostatic function in the musculoskeletal system. Patients affected by Robinow Syndrome, a condition characterised by several dysmorphic features associated with skeletal dysplasia, bear mutations in either Wnt5a or ROR2 genes.^44^, ^45^, ^46^ Studies in vivo showed that Wnt5a drives limb elongation in a concentration‐dependent manner during development by activating the Planar Cell Polarity (PCP) pathway in chondrocytes.^47^ As for the Wnt/β‐catenin pathway, the ROR2 pathway also seems to get re‐activated in the AC in OA: the expression of Wnt5a and of the ROR2 targets Yes‐associated protein (YAP) and connective growth factor (CTGF) are upregulated in the AC of OA patients. ROR2 blockade supported an anabolic response in the AC in an in vivo model of OA, although through Wnt‐independent mechanisms.^48^

The Wnt/CaMKII pathway has also been shown to have a pivotal role in bone and cartilage development. Studies in chicken and mice showed that activation of CaMKII promotes cartilage hypertrophic differentiation during limb formation.^49^, ^50^ Several studies showed that CaMKII is activated in OA, both in human and in animal models of the disease.^51^, ^52^ We have recently shown that pharmacological blockade of CaMKII can exacerbate cartilage damage in a murine model of OA.^51^ This is in keeping with a recent in vitro study showing that inhibition of CaMKII can decrease the anabolic activity of bone morphogenetic proteins 2 and 4 on the synthesis of ECM components in isolated chondrocytes^53^ but it is in contrast with previous work from the Saito's group showing that CaMKII can promote OA development in mice via activation of the Hes1 transcription factor.^52^

## 7 β‐CATENIN‐DEPENDENT AND ‐INDEPENDENT PATHWAYS: A MATTER OF BALANCE

What is then the role of the Wnt signalling in the AC and OA? Can it be considered as a therapeutic target for this disease? An answer to this question comes from the recent discovery of the first molecule classified as a disease‐modifying drug for the treatment of OA, SM04690, now known under the commercial name of Lorecivivint. Lorecivivint, which is currently in Phase III clinical trial, is an inhibitor of Cdc2‐like kinase (CLK1), an enzyme‐regulating splicing events, and blocks the activation of the Wnt signalling at transcriptional level. Nonetheless, the drug also inhibits the Dual‐specificity tyrosine phosphorylation‐regulated kinase 1A, which modulates the inflammatory response.^54^

The discovery of this drug highlights how our understanding of the molecular events, intrinsic and extrinsic to the pathway, is still relatively poor and that the lack of this information is potentially dampening the development of additional therapeutics. Integrative approaches, aimed at investigating the simultaneous modulation of multiple molecules, will therefore be required to understand how tissue homeostasis is maintained and could be re‐established once disrupted.

Our recent work contributed to unravel the complexity of the regulation of the Wnt signalling in the AC. We showed that a single ligand, Wnt3a, could promote both intracellular accumulation of β‐catenin and intracellular activation of CaMKII in the AC, thus simultaneously activating β‐catenin‐dependent and ‐independent branches of the Wnt signalling. Critically, while the first increases proportionally to Wnt3a concentration and drives proliferation, the second response is higher when chondrocytes are stimulated with low concentrations of Wnt3a and drives phenotypic changes.^23^

These two physiological outcomes are in a steady‐state equilibrium under normal conditions and are reciprocally inhibitory, leading to the hypothesis  that signalling pathway interactions must be considered to understand how homeostasis is maintained in the tissue. This concept has recently been extrapolated to describe the interaction among the separate branches of the signalling in mathematical terms.^55^

Our more recent data further validated this hypothesis by showing that while the Wnt/CaMKII pathway is also upregulated in OA in vivo, its pharmacological inhibition exacerbated cartilage degradation in a murine model of the disease.^51^

Wnt3a has been shown to mediate the simultaneous activation of β‐catenin‐dependent and Ca^2+^‐dependent pathways in other biological systems^24^, ^56^, ^57^ which have been shown to be reciprocally regulatory.^58^

Separately, we showed that Wnt16 also shares the ability of mediating multiple and contrasting signals in the cartilage.^43^ Despite promoting anabolic effects through the activation of the Wnt/β‐catenin pathway, Wnt16 antagonises the activation of this signalling cascade when induced by Wnt3a, therefore avoiding the activation of the pathway over a certain threshold.^43^

**FIGURE 1:**
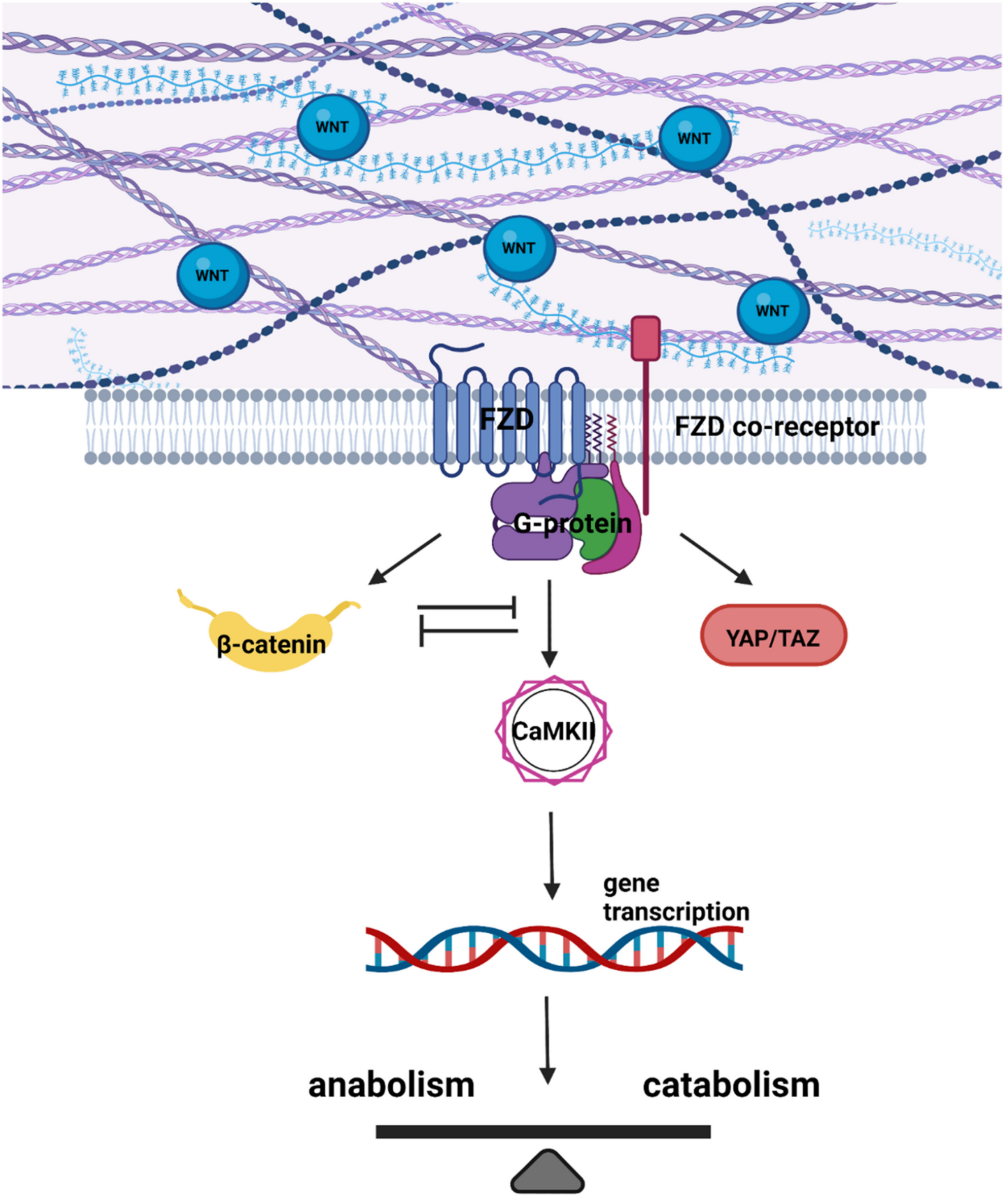
Schematic representation illustrating the importance of three branches of the Wnt‐signalling network in the maintenance of cartilage homeostasis.

Taken together, these data strengthen the argument that a fine‐tuned balance in the activation of the Wnt signalling is required to maintain cartilage homeostasis and that buffering mechanisms ‐ for example competitive antagonism of ligands for the same receptors or epigenetic modifications ‐ are in place to avoid prolonged perturbation of this equilibrium, as when this occurs, it can lead to tissue degeneration and OA development  (Figure 1).

## 8 MAINTAINING THE BALANCE: THE ROLE OF RECEPTORS AND CO‐RECEPTORS

Ligands and receptors are important gatekeepers of the activation of the different Wnt pathways. Beyond Wnt3a, Wnt5a and Wnt7a have been reported to activate β‐catenin‐dependent and ‐independent pathways^59^, ^60^, ^61^ in a context‐specific manner.

Engagement of Wnt ligands with different isoforms of FZDs is linked to the activation of both β‐catenin‐dependent and ‐independent pathways. Interaction of FZDs with different co‐receptors is also considered pivotal in determining which branch of the network is going to be activated. Nonetheless, a detailed characterisation of the specificity of binding of different ligands for different isoforms, as well as how the availability in the extracellular environment of other Wnts can influence their affinity and specificity of binding, remains unclear for most tissues and primary cells.

The generation of fluorescence and luminescence‐based biosensors has allowed some progresses in this direction.^62^ Bourhis and colleagues demonstrated that in insect cells, individual Wnts can simultaneously bind to different domains of LRP6. They also showed that Wnt5a, Wnt5b and Wnt3a can all bind to FZD8. While simultaneous interactions for the same receptors are possible, the affinity of the different ligands for the different isoforms and binding sites can vary. Therefore, the binding scenario and the activation of different intracellular signalling can indeed be different depending on the availability of different ligands in the extracellular space.^63^

Furthermore, also the availability of FZD on the cell surface can be influenced by external factors: R‐spondins, Wnt agonists binding to leucine‐rich repeat‐containing G‐protein‐coupled receptors (LGRs), decrease the ubiquitination of FZD receptors, reducing their turnover and therefore potentiating the activation of the Wnt/β‐catenin pathway.^64^

Finally, the activity and availability of different co‐receptors such as LRP‐5 and 6 can also add up to the complexity of this signalling network. Studies in vivo showed that deficiency of LRP5 can exacerbate or decrease degeneration of the AC in murine models of osteoarthritis, in a model‐dependent way.^65^, ^66^ This suggests that external factors, such as biomechanics and inflammation, can also alter the signalling response, through vastly uncharacterised mechanisms.

Frizzled are a family of seven‐span transmembrane G‐coupled receptors.^67^

G protein‐coupled‐receptor are not solely limited to activating and initiating their own downstream signalling pathways; they are also able to communicate with each other, influencing and mediating the activation and subsequent downstream signalling of one another. This can occur through synergistic crosstalk interactions between either different signalling transduction pathways, through either the same/different classes of GPCRs or through competitive antagonism, whereby they are able to communicate and prevent their own signal transduction pathway.^68^ As a result, this can influence the potency and efficacy of the signalling, which, at times, can resemble behaviour that is typically observed with allosteric interactions.

Crosstalk can occur between GPCRs coupled to different G proteins, enhancing the cellular response of one of the pathways. This could be interactions between G_i_‐ and G_q_‐coupled receptors or G_s_‐ and G_q_‐coupled receptors. In the case of smooth muscle contraction, crosstalk occurs between G_i_‐ and G_q_‐coupled receptors.^68^ When there is no crosstalk among GPCRs and their downstream signalling, noradrenaline activates the G_q_‐coupled α‐adrenergic receptors, resulting in smooth muscle contractions. However, in the presence of Neuropeptide Y (NPY), a co‐transmitter that activates the G_i_‐coupled Neuropeptide Y receptors, crosstalk initiates with α‐adrenergic receptors, enhancing the smooth muscle contractions. An advantage to this augmented response is that lower concentrations of noradrenaline are thus required to elicit smooth muscle contraction when co‐administered with NPY.^68^

Synergistic interactions can also occur between different classes of GPCRs. The Smoothened Receptor (SMO) is a Class F GPCR that, upon activation, mediates Sonic Hedgehog (SHH) signalling. Pusapati et al showed, using CRISPR experiments, that the sensitivity of NIH/3 T3 fibroblasts and spinal neural progenitors to SHH is increased when there is a loss of GPR161, an orphan Class A GPCR (Pusapati et al., 2018). It is important to note that the signalling still depends on the SMO receptor, however, through increasing sensitivity to the morphogen SHH. This shows the unique manner in which GPR161 is able to interact with the SMO receptor, influencing its subsequent signalling.^69^

Finally, Civciristov and colleagues demonstrated that GPCRs can also respond differently to high and low concentrations of the same ligands. The response to low concentrations of the ligands was spatially and temporally distinct and resulted in different intracellular proteomic profiles. This was due to the preassembly of GPCR high‐order complexes to the plasma membrane.^70^ The characterisation of how the activation of the Wnt signalling is mediated at receptor level in the AC and in musculoskeletal tissues remains vastly unknown. While this presents multiple challenges, it has, however, the potential to allow the development of new therapeutic strategies to re‐establish tissue homeostasis and halt OA progression.

## 9 CONCLUSIONS

Several intracellular mechanisms have been shown to be key in maintaining the right threshold of activation of the different branches of the Wnt signalling network.^71^, ^72^ Some of these have been reviewed recently by us and others in other reviews^73^, ^74^, ^75^, ^76^ and will also be discussed in future publications.

The overall message of this review is the consideration that a change of mindset in the way we consider signalling pathways as a pharmacological target for OA and other diseases is needed. As already highlighted by Amerongen and Nusse in 2009,^77^ we should stop considering the Wnt signalling as a group of individual and linear signalling cascades but rather start considering them as a network, whose branches interact with each other and reciprocally influence their activation or repression state. It is also clear that we should not define ligand/receptor interaction as absolute concepts, but rather we should validate them within specific biological contexts taking in account and, when possible, mimicking, the extracellular environment characterising different tissues in physiology and disease. System biology approaches and mathematical models will become essential tools to be used to clarify these complicated interactions.

Clarifying how the balance of this intricate network is maintained will be pivotal for the discovery of new therapeutic targets, where the treatment will probably not focus anymore on individual molecules but on the re‐establishment of the overall signalling homeostasis.

## CONFLICT OF INTEREST STATEMENT

The authors declare no conflict of interest.

## References

[1] Cui A , Li H , Wang D , Zhong J , Chen Y , Lu H . Global, regional prevalence, incidence and risk factors of knee osteoarthritis in population‐based studies. EClinicalMedicine. 2020; 29‐30:100587 10.1016/j.eclinm.2020.100587 PMC7704420 34505846

[2] Goldring SR , Goldring MB . Changes in the osteochondral unit during osteoarthritis: structure, function and cartilage bone crosstalk. Nat Rev Rheumatol. 2016;12:632‐644. 27652499 10.1038/nrrheum.2016.148

[3] Xie DX , Wei J , Zeng C , et al. Association between metabolic syndrome and knee osteoarthritis: a cross‐sectional study. BMC Musculoskelet Disord. 2017;18:1‐7. 29246142 10.1186/s12891-017-1890-9 PMC5732466

[4] Puig‐Junoy J , Ruiz Zamora A . Socio‐economic costs of osteoarthritis: a systematic review of cost‐of‐illness studies. Semin Arthritis Rheum. 2015;44:531‐541. 25511476 10.1016/j.semarthrit.2014.10.012

[5] Nuesch E , Dieppe P , Reichenbach S , Williams S , Iff S , Juni P . All cause and disease specific mortality in patients with knee or hip osteoarthritis: population based cohort study. BMJ. 2011;342:d1165. 21385807 10.1136/bmj.d1165 PMC3050438

[6] Ferket BS , Feldman Z , Zhou J , Oei EH , Bierma‐Zeinstra SMA , Mazumdar M . Impact of total knee replacement practice: cost effectiveness analysis of data from the osteoarthritis initiative. BMJ. 2017;356:j1131. 28351833 10.1136/bmj.j1131 PMC6284324

[7] Sophia Fox AJ , Bedi A , Rodeo SA . The basic science of articular cartilage: structure, composition, and function. Sports Health. 2009;1:461‐468. 23015907 10.1177/1941738109350438 PMC3445147

[8] Archer CW , Francis‐West P . The chondrocyte. Int J Biochem Cell Biol. 2003;35:401‐404. 12565700 10.1016/s1357-2725(02)00301-1

[9] Malinin T , Ouellette EA . Articular cartilage nutrition is mediated by subchondral bone: a long‐term autograft study in baboons. Osteoarthr Cartil. 2000;8:483‐491. 10.1053/joca.1999.0324 11069733

[10] Troeberg L , Nagase H . Proteases involved in cartilage matrix degradation in osteoarthritis. Biochim Biophys Acta. 2012;1824:133‐145. 21777704 10.1016/j.bbapap.2011.06.020 PMC3219800

[11] Yamamoto K , Wilkinson D , Bou‐Gharios G . Targeting dysregulation of metalloproteinase activity in osteoarthritis. Calcif Tissue Int. 2020;1093(109):277‐290. 10.1007/s00223-020-00739-7 PMC8403128 32772139

[12] Yang L , Tsang KY , Tang HC , Chan D , Cheah KSE . Hypertrophic chondrocytes can become osteoblasts and osteocytes in endochondral bone formation. Proc Natl Acad Sci U S A. 2014;111:12097‐12102. 25092332 10.1073/pnas.1302703111 PMC4143064

[13] Zhou X , von der Mark K , Henry S , Norton W , Adams H , de Crombrugghe B . Chondrocytes transdifferentiate into osteoblasts in endochondral bone during development, postnatal growth and fracture healing in mice. PLoS Genet. 2014;10:e1004820. 25474590 10.1371/journal.pgen.1004820 PMC4256265

[14] Yang G , Zhu L , Hou N , et al. Osteogenic fate of hypertrophic chondrocytes. Cell Res. 2014;2410(24):1266‐1269. 10.1038/cr.2014.111 PMC4185343 25145361

[15] Usami Y , Gunawardena AT , Iwamoto M , Enomoto‐Iwamoto M . Wnt signaling in cartilage development and diseases: lessons from animal studies. Lab Invest. 2016;96:186‐196. 26641070 10.1038/labinvest.2015.142 PMC4838282

[16] Goldring MB , Marcu KB . Cartilage homeostasis in health and rheumatic diseases. Arthritis Res Ther. 2009;11:224. 19519926 10.1186/ar2592 PMC2714092

[17] Stamos JL , Weis WI . The β‐catenin destruction complex. Cold Spring Harb Perspect Biol. 2013;5(1):a007898. 23169527 10.1101/cshperspect.a007898 PMC3579403

[18] Clevers H , Nusse R . Wnt/β‐catenin signaling and disease. Cell. 2012;149:1192‐1205. 22682243 10.1016/j.cell.2012.05.012

[19] Kishida S , Yamamoto H , Ikeda S , et al. Axin, a negative regulator of the wnt signaling pathway, directly interacts with adenomatous polyposis coli and regulates the stabilization of beta‐catenin. J Biol Chem. 1998;273:10823‐10826. 9556553 10.1074/jbc.273.18.10823

[20] Shi F , Mendrola JM , Sheetz JB , et al. ROR and RYK extracellular region structures suggest that receptor tyrosine kinases have distinct WNT‐recognition modes. Cell Rep. 2021;37:109834. 34686333 10.1016/j.celrep.2021.109834 PMC8650758

[21] Davidson G . LRPs in WNT Signalling. Handb Exp Pharmacol. 2021;269:45‐73. 34490514 10.1007/164_2021_526

[22] Kühl M , Sheldahl LC , Malbon CC , Moon RT . Ca(2+)/calmodulin‐dependent protein kinase II is stimulated by Wnt and frizzled homologs and promotes ventral cell fates in Xenopus. J Biol Chem. 2000;275:12701‐12711. 10777564 10.1074/jbc.275.17.12701

[23] Nalesso G , Sherwood J , Bertrand J , et al. WNT‐3A modulates articular chondrocyte phenotype by activating both canonical and noncanonical pathways. J Cell Biol. 2011;193:551‐564. 21536751 10.1083/jcb.201011051 PMC3087013

[24] Weivoda MM , Ruan M , Hachfeld CM , et al. Wnt signaling inhibits osteoclast differentiation by activating canonical and noncanonical cAMP/PKA pathways. J Bone Miner Res. 2016;31:65‐75. 26189772 10.1002/jbmr.2599 PMC4758681

[25] Martineau X , Abed É , Martel‐Pelletier J , Pelletier JP , Lajeunesse D . Alteration of Wnt5a expression and of the non‐canonical Wnt/PCP and Wnt/PKC‐Ca2+ pathways in human osteoarthritis osteoblasts. PLoS One. 2017;12(8):e0180711. 28777797 10.1371/journal.pone.0180711 PMC5544184

[26] Guo X , Day TF , Jiang X , Garrett‐Beal L , Topol L , Yang Y . Wnt/beta‐catenin signaling is sufficient and necessary for synovial joint formation. Genes Dev. 2004;18:2404‐2417. 15371327 10.1101/gad.1230704 PMC522990

[27] Später D , Hill TP , Gruber M , Hartmann C . Role of canonical Wnt‐signalling in joint formation. Eur Cell Mater. 2006;12:71‐80. 17115376 10.22203/ecm.v012a09

[28] Xuan F , Yano F , Mori D , et al. Wnt/β‐catenin signaling contributes to articular cartilage homeostasis through lubricin induction in the superficial zone. Arthritis Res Ther. 2019;21:247. 31771658 10.1186/s13075-019-2041-5 PMC6880374

[29] Loughlin J . Polymorphism in signal transduction is a major route through which osteoarthritis susceptibility is acting. Curr Opin Rheumatol. 2005;17:629‐633. 16093844 10.1097/01.bor.0000176687.85198.49

[30] Rodriguez‐Lopez J , Pombo‐Suarez M , Liz M , Gomez‐Reino JJ , Gonzalez A . Further evidence of the role of frizzled‐related protein gene polymorphisms in osteoarthritis. Ann Rheum Dis. 2007;66:1052‐1055. 17237116 10.1136/ard.2006.065938 PMC1954696

[31] Baker‐LePain JC , Lynch JA , Parimi N , et al. Variant alleles of the Wnt antagonist FRZB are determinants of hip shape and modify the relationship between hip shape and osteoarthritis. Arthritis Rheum. 2012;64:1457‐1465. 22544526 10.1002/art.34526 PMC3340442

[32] Thysen S , Luyten FP , Lories RJ . Loss of Frzb and Sfrp1 differentially affects joint homeostasis in instability‐induced osteoarthritis. Osteoarthr Cartil. 2015;23:275‐279. 10.1016/j.joca.2014.10.010 25450854

[33] Lories RJ , Peeters J , Bakker A , et al. Articular cartilage and biomechanical properties of the long bones in Frzb‐knockout mice 1. Arthritis Rheum. 2007;56:4095‐4103. 18050203 10.1002/art.23137

[34] Leijten JCH , Bos SD , Landman EBM , et al. GREM1, FRZB and DKK1 mRNA levels correlate with osteoarthritis and are regulated by osteoarthritis‐associated factors. Arthritis Res Ther. 2013;15(5):R126. 24286177 10.1186/ar4306 PMC3978825

[35] Dell'accio F , De Bari C , Eltawil NM , Vanhummelen P , Pitzalis C . Identification of the molecular response of articular cartilage to injury, by microarray screening: Wnt‐16 expression and signaling after injury and in osteoarthritis. Arthritis Rheum. 2008;58:1410‐1421. 18438861 10.1002/art.23444

[36] Rhee J , Ryu JH , Kim JH , Chun CH , Chun JS . α‐Catenin inhibits β‐catenin‐T‐cell factor/lymphoid enhancing factor transcriptional activity and collagen type II expression in articular chondrocytes through formation of Gli3R.α‐catenin.β‐catenin ternary complex. J Biol Chem. 2012;287:11751‐11760. 22298781 10.1074/jbc.M111.281014 PMC3320923

[37] Yuasa T , Otani T , Koike T , Iwamoto M , Enomoto‐Iwamoto M . Wnt/beta‐catenin signaling stimulates matrix catabolic genes and activity in articular chondrocytes: its possible role in joint degeneration. Lab Invest. 2008;88:264‐274. 18227807 10.1038/labinvest.3700747

[38] Hui T , Zhou Y , Wang T , et al. Activation of β‐catenin signaling in aggrecan‐expressing cells in temporomandibular joint causes osteoarthritis‐like defects. Int J Oral Sci. 2018;102(10):1‐8. 10.1038/s41368-018-0016-z PMC5966811 29686224

[39] Zhu M , Tang D , Wu Q , et al. Activation of beta‐catenin signaling in articular chondrocytes leads to osteoarthritis‐like phenotype in adult beta‐catenin conditional activation mice. J Bone Miner Res. 2009;24:12‐21. 18767925 10.1359/JBMR.080901 PMC2640321

[40] Cai S , Zou Y , Zhao Y , et al. Mechanical stress reduces secreted frizzled‐related protein expression and promotes temporomandibular joint osteoarthritis via Wnt/β‐catenin signaling. Bone. 2022;161:116445. 35589066 10.1016/j.bone.2022.116445

[41] Chen M , Zhu M , Awad H , et al. Inhibition of beta‐catenin signaling causes defects in postnatal cartilage development. J Cell Sci. 2008;121:1455‐1465. 18397998 10.1242/jcs.020362 PMC2636704

[42] Zhu M , Chen M , Zuscik M , et al. Inhibition of beta‐catenin signaling in articular chondrocytes results in articular cartilage destruction. Arthritis Rheum. 2008;58:2053‐2064. 18576323 10.1002/art.23614 PMC2667964

[43] Nalesso G , Thomas BL , Sherwood JC , et al. WNT16 antagonises excessive canonical WNT activation and protects cartilage in osteoarthritis. Ann Rheum Dis. 2016;76(1):218‐226. doi:10.1136/annrheumdis-2015-208577 27147711 PMC5264226

[44] Person AD , Beiraghi S , Sieben CM , et al. WNT5A mutations in patients with autosomal dominant Robinow syndrome. Dev Dyn. 2010;239:327. 19918918 10.1002/dvdy.22156 PMC4059519

[45] Van Bokhoven H , Celli J , Kayserili H , et al. Mutation of the gene encoding the ROR2 tyrosine kinase causes autosomal recessive Robinow syndrome. Nat Genet. 2000;25:423‐426. 10932187 10.1038/78113

[46] Afzal AR , Rajab A , Fenske CD , et al. Recessive Robinow syndrome, allelic to dominant brachydactyly type B, is caused by mutation of ROR2. Nat Genet. 2000;25:419‐422. 10932186 10.1038/78107

[47] Andre P , Wang Q , Wang N , et al. The Wnt coreceptor Ryk regulates Wnt/planar cell polarity by modulating the degradation of the core planar cell polarity component Vangl2. J Biol Chem. 2012;287:44518‐44525. 23144463 10.1074/jbc.M112.414441 PMC3531765

[48] Thorup AS , Strachan D , Caxaria S , et al. ROR2 blockade as a therapy for osteoarthritis. Sci Transl Med. 2020;12(561):eaax3063. 32938794 10.1126/scitranslmed.aax3063

[49] Taschner MJ , Rafigh M , Lampert F , Schnaiter S , Hartmann C . Ca2+/Calmodulin‐dependent kinase II signaling causes skeletal overgrowth and premature chondrocyte maturation. Dev Biol. 2008;317:132‐146. 18342847 10.1016/j.ydbio.2008.02.007

[50] Li Y , Ahrens MJ , Wu A , Liu J , Dudley AT . Calcium/calmodulin‐dependent protein kinase II activity regulates the proliferative potential of growth plate chondrocytes. Development. 2011;138:359‐370. 21177348 10.1242/dev.052324 PMC3005607

[51] Nalesso G , Thorup AS , Eldridge SE , et al. Calcium calmodulin kinase II activity is required for cartilage homeostasis in osteoarthritis. Sci Rep. 2021;11:5682. 33707504 10.1038/s41598-021-82067-w PMC7952598

[52] Sugita S , Hosaka Y , Okada K , et al. Transcription factor Hes1 modulates osteoarthritis development in cooperation with calcium/calmodulin‐dependent protein kinase 2. Proc Natl Acad Sci. 2015;112:3080‐3085. 25733872 10.1073/pnas.1419699112 PMC4364241

[53] Saitta B , Elphingstone J , Limfat S , Shkhyan R , Evseenko D . CaMKII inhibition in human primary and pluripotent stem cell‐derived chondrocytes modulates effects of TGFβ and BMP through SMAD signaling. Osteoarthr Cartil. 2018;27:158‐171. doi:10.1016/j.joca.2018.08.017 PMC6309757 30205161

[54] Yazici Y , McAlindon TE , Gibofsky A , et al. Lorecivivint, a novel Intraarticular CDC‐like kinase 2 and dual‐specificity tyrosine phosphorylation‐regulated kinase 1A inhibitor and Wnt pathway modulator for the treatment of knee osteoarthritis: a phase II randomized trial. Arthritis Rheumatol. 2020;72:1694‐1706. 32432388 10.1002/art.41315 PMC7589351

[55] Sträng JE , Schuler R , Kühl M , Kestler HA . Switch‐like behavior enables Wnt11 concentration specific response during dorso‐ventral axis formation in Xenopus laevis. J Theor Biol. 2017;429:82‐94. 28648560 10.1016/j.jtbi.2017.06.027

[56] Flores‐Hernández E , Velázquez DM , Castañeda‐Patlán MC , et al. Canonical and non‐canonical Wnt signaling are simultaneously activated by Wnts in colon cancer cells. Cell Signal. 2020;72:109636. 32283254 10.1016/j.cellsig.2020.109636

[57] Avila ME , Sepúlveda FJ , Burgos CF , et al. Canonical Wnt3a modulates intracellular calcium and enhances excitatory neurotransmission in hippocampal neurons. J Biol Chem. 2010;285:18939‐18947. 20404321 10.1074/jbc.M110.103028 PMC2881816

[58] Narendra Talabattula VA , Morgan P , Frech MJ , et al. Non‐canonical pathway induced by Wnt3a regulates β‐catenin via Pyk2 in differentiating human neural progenitor cells. Biochem Biophys Res Commun. 2017;491:40‐46. 28694190 10.1016/j.bbrc.2017.07.030

[59] Gibson AL , Hui Mingalone CK , Foote AT , Uchimura T , Zhang M , Zeng L . Wnt7a inhibits IL‐1β induced catabolic gene expression and prevents articular cartilage damage in experimental osteoarthritis. Sci Rep. 2017;7:41823. 28165497 10.1038/srep41823 PMC5292965

[60] Mikels AJ , Nusse R . Purified Wnt5a protein activates or inhibits β‐catenin–TCF signaling depending on receptor context. PLoS Biol. 2006;4:e115. 16602827 10.1371/journal.pbio.0040115 PMC1420652

[61] Van Amerongen R , Fuerer C , Mizutani M , Nusse R . Wnt5a can both activate and repress Wnt/β‐catenin signaling during mouse embryonic development. Dev Biol. 2012;369:101‐114. 22771246 10.1016/j.ydbio.2012.06.020 PMC3435145

[62] Kozielewicz P , Shekhani R , Moser S , et al. Quantitative profiling of WNT‐3A binding to all human frizzled paralogues in HEK293 cells by NanoBiT/BRET assessments. ACS Pharmacol Transl Sci. 2021;4:1235‐1245. 34151213 10.1021/acsptsci.1c00084 PMC8205236

[63] Bourhis E , Tam C , Franke Y , et al. Reconstitution of a frizzled8.Wnt3a.LRP6 signaling complex reveals multiple Wnt and Dkk1 binding sites on LRP6. J Biol Chem. 2010;285:9172‐9179. 20093360 10.1074/jbc.M109.092130 PMC2838336

[64] Knight MN , Hankenson KD . R‐spondins: novel matricellular regulators of the skeleton. Matrix Biol. 2014;37:157‐161. 24980904 10.1016/j.matbio.2014.06.003

[65] Lodewyckx L , Luyten FP , Lories RJ . Genetic deletion of low‐density lipoprotein receptor‐related protein 5 increases cartilage degradation in instability‐induced osteoarthritis. Rheumatology (Oxford). 2012;51:1973‐1978. 22850184 10.1093/rheumatology/kes178

[66] Shin Y , Huh Y , Kim K , et al. Low‐density lipoprotein receptor‐related protein 5 governs Wnt‐mediated osteoarthritic cartilage destruction. Arthritis Res Ther. 2014;16:R37. 24479426 10.1186/ar4466 PMC3978879

[67] Sun Y , Wang W , Zhao C . Frizzled receptors in tumors, focusing on signaling, roles, modulation mechanisms, and targeted therapies. Oncol Res. 2021;28:661. 32998794 10.3727/096504020X16014648664459 PMC7962935

[68] Selbie LA , Hill SJ . G protein‐coupled‐receptor cross‐talk: the fine‐tuning of multiple receptor‐signalling pathways. Trends Pharmacol Sci. 1998;19:87‐93. 9584624 10.1016/s0165-6147(97)01166-8

[69] Pusapati GV , Kong JH , Patel BB , et al. G protein‐coupled receptors control the sensitivity of cells to the morphogen sonic hedgehog. Sci Signal. 2018;11(516):eaao5749. 29438014 10.1126/scisignal.aao5749 PMC5828112

[70] Civciristov S , Ellisdon AM , Suderman R , et al. Preassembled GPCR signaling complexes mediate distinct cellular responses to ultralow ligand concentrations. Sci Signal. 2018;11(551):eaan1188. 30301787 10.1126/scisignal.aan1188 PMC7416780

[71] Topol L , Jiang X , Choi H , Garrett‐Beal L , Carolan PJ , Yang Y . Wnt‐5a inhibits the canonical Wnt pathway by promoting GSK‐3‐independent beta‐catenin degradation. J Cell Biol. 2003;162:899‐908. 12952940 10.1083/jcb.200303158 PMC2172823

[72] Park HW , Kim YC , Yu B , et al. Alternative Wnt signaling activates YAP/TAZ. Cell. 2015;162:780‐794. 26276632 10.1016/j.cell.2015.07.013 PMC4538707

[73] Chun JS , Oh H , Yang S , Park M . Wnt signaling in cartilage development and degeneration. BMB Rep. 2008;41:485‐494. 18682032 10.5483/bmbrep.2008.41.7.485

[74] Cherifi C , Monteagudo S , Lories RJ . Promising targets for therapy of osteoarthritis: a review on the Wnt and TGF‐β signalling pathways. Ther Adv Musculoskelet Dis. 2021;13:1759720X2110069. 10.1177/1759720X211006959 PMC8053758 33948125

[75] Monteagudo S , Lories RJ . Cushioning the cartilage: a canonical Wnt restricting matter. Nat Rev Rheumatol. 2017;13:670‐681. 29021569 10.1038/nrrheum.2017.171

[76] De Palma A , Nalesso G . WNT Signalling in osteoarthritis and its pharmacological targeting. Handb Exp Pharmacol. 2021;269:337‐356. 34510305 10.1007/164_2021_525

[77] van Amerongen R , Nusse R . Towards an integrated view of Wnt signaling in development. Development. 2009;136:3205‐3214. 19736321 10.1242/dev.033910

